# Positional Dynamics and Glycosomal Recruitment of Developmental Regulators during Trypanosome Differentiation

**DOI:** 10.1128/mBio.00875-19

**Published:** 2019-07-09

**Authors:** Balázs Szöőr, Dorina V. Simon, Federico Rojas, Julie Young, Derrick R. Robinson, Timothy Krüger, Markus Engstler, Keith R. Matthews

**Affiliations:** a Institute for Immunology and Infection Research, School of Biological Sciences, University of Edinburgh, Edinburgh, United Kingdom; b CNRS, Microbiology Fundamental and Pathogenicity, MFP UMR 5234, University of Bordeaux, Bordeaux, France; c Cell and Developmental Biology, University of Würzburg Biozentrum, Würzburg, Germany; Yale University School of Public Health

**Keywords:** development, differentiation, organelle, parasite, trypanosome, glycosome

## Abstract

Glycosomes are peroxisome-related organelles that compartmentalize the glycolytic enzymes in kinetoplastid parasites. These organelles are developmentally regulated in their number and composition, allowing metabolic adaptation to the parasite’s needs in the blood of mammalian hosts or within their arthropod vector. A protein phosphatase cascade regulates differentiation between parasite developmental forms, comprising a tyrosine phosphatase, Trypanosoma brucei PTP1 (TbPTP1), which dephosphorylates and inhibits a serine threonine phosphatase, TbPIP39, which promotes differentiation. When TbPTP1 is inactivated, TbPIP39 is activated and during differentiation becomes located in glycosomes. Here we have tracked TbPIP39 recruitment to glycosomes during differentiation from bloodstream “stumpy” forms to procyclic forms. Detailed microscopy and live-cell imaging during the synchronous transition between life cycle stages revealed that in stumpy forms, TbPIP39 is located at a periflagellar pocket site closely associated with TbVAP, which defines the flagellar pocket endoplasmic reticulum. TbPTP1 is also located at the same site in stumpy forms, as is REG9.1, a regulator of stumpy-enriched mRNAs. This site provides a molecular node for the interaction between TbPTP1 and TbPIP39. Within 30 min of the initiation of differentiation, TbPIP39 relocates to glycosomes, whereas TbPTP1 disperses to the cytosol. Overall, the study identifies a “stumpy regulatory nexus” (STuRN) that coordinates the molecular components of life cycle signaling and glycosomal development during transmission of Trypanosoma brucei.

## Abstract (precis)

African trypanosomes are parasites of sub-Saharan Africa responsible for both human and animal disease. The parasites are transmitted by tsetse flies, and completion of their life cycle involves progression through several development steps. The initiation of differentiation between blood and tsetse fly forms is signaled by a phosphatase cascade, ultimately trafficked into peroxisome-related organelles called glycosomes that are unique to this group of organisms. Glycosomes undergo substantial remodeling of their composition and function during the differentiation step, but how this is regulated is not understood. Here we identify a cytological site where the signaling molecules controlling differentiation converge before the dispersal of one of them into glycosomes. In combination, the study provides the first insight into the spatial coordination of signaling pathway components in trypanosomes as they undergo cell-type differentiation.

## INTRODUCTION

The dynamic regulation of organelle biogenesis or composition often involves intimate contact between the endoplasmic reticulum (ER) and the membrane of the target organelle (1). This enables the maturation and modification of organellar protein content, influencing mitochondrial, Golgi, or peroxisomal components, whereas interorganellar contacts can also contribute to signaling events within cells, bringing regulatory molecules into proximity or trafficking them for degradation (2). The protein contacts at the interface between organelles are often diverse and characteristic of each organellar type, predominantly interacting with vesicle-associated membrane protein (VAMP)-associated proteins (VAPs) on the ER membrane.

In addition to the conventional organelles typical of eukaryotic cells, evolutionarily divergent kinetoplastid parasites are characterized by their possession of glycosomes, specialist organelles that harbor the enzymes of glycolysis (3). Although unique in their compartmentation of glycolytic enzymes, glycosomes are related to peroxisomes, sharing with those organelles a similar (though divergent) machinery for import, insertion of membrane proteins (PEX16 and PEX19), and peroxisome proliferation and membrane curvature, as well as their capacity for either lipid biosynthesis or purine and pyrimidine biosynthesis (4). Glycosomes are also dynamic in composition and number in response to the metabolic demands of the parasite, their synthesis and turnover involving biogenesis and degradation mechanisms similar to those of the peroxisomes of yeast and mammalian cells. This capacity for biosynthesis and turnover enables peroxisomes and glycosomes to exploit different nutrient conditions or adapt to different developmental forms.

Kinetoplastid parasites comprise pathogens of mammals that are frequently transmitted by arthropod vectors. Among the best characterized and tractable are the African trypanosomes of Trypanosoma brucei spp. These parasites live extracellularly in the bloodstream and tissues of mammalian hosts, where they cause human sleeping sickness and the livestock disease nagana (5, 6). Trypanosomes are spread by blood-feeding tsetse flies, the passage between the blood and the insect gut involving a switch from a glucose-based energy metabolism to one reliant on amino acids (7). Pivotal to the successful colonization of the tsetse fly are so-called “stumpy forms,” quiescent bloodstream forms that show several adaptations for survival upon uptake by tsetse flies (8), including partial elaboration of their mitochondrion in preparation for the switch from glucose-dependent energy generation via glycolysis (9–12). Stumpy forms arise from proliferative slender forms in the bloodstream in a quorum-sensing response dependent upon parasite density (13). This results in the accumulation of uniform populations of stumpy forms that are cell cycle arrested in G_1_/G_0_ and sensitized for differentiation when taken up in a tsetse fly blood meal (14), this culminating in the production of a population of differentiated procyclic forms that colonize the tsetse midgut. The same transition can also be enacted *in vitro* by exposing stumpy forms to reduced temperature and *cis*-aconitate/citrate, which generates a highly synchronized differentiation model allowing cytological events to be readily tracked and quantitated in the population (15).

The signaling events that stimulate the differentiation of stumpy forms to procyclic forms are quite well characterized. Thus, stumpy forms are held poised for differentiation by the action of a negative regulator of differentiation, T. brucei PTP1 (TbPTP1), a tyrosine-specific phosphatase (16, 17). A substrate of TbPTP1 is the DxDxT/V class serine threonine phosphatase T. brucei PIP39 (TbPIP39), which is dephosphorylated on tyrosine 278 by TbPTP1, this interaction reducing the activity of TbPIP39 and so preventing differentiation (18). When exposed to reduced temperature, as would occur during a tsetse fly blood meal (19), blood citrate is transported by “PAD” proteins whose expression is elevated on stumpy forms at 20°C (20). When exposed to citrate/*cis*-aconitate, TbPTP1 is inactivated and TbPIP39 becomes phosphorylated and activated, thus stimulating differentiation of the parasites. Interestingly, sequence analysis of TbPIP39 revealed the presence of a PTS1 glycosomal localization motif (-SRL), and this localization was confirmed in procyclic forms by both its colocalization with glycosomal markers (17) and its detection in glycosomal proteome analysis (21). This linked differentiation signaling in the bloodstream-form parasites with glycosomal signaling during differentiation, with TbPIP39 being expressed in stumpy forms but not slender forms and being localized in glycosomes in procyclic forms.

Here we have exploited the differential expression and glycosomal location of TbPIP39 to explore the spatial positioning of differentiation signaling molecules during the transformation of stumpy forms to procyclic forms. Our results reveal the coincidence of TbPIP39 and TbPTP1 in bloodstream stumpy forms at a novel periflagellar pocket location, closely associated with a flagellar pocket ER contact site defined by T. brucei VAP (TbVAP) (22). This provides a novel signaling response linking environmental perception with organellar dynamics during the developmental cycle of the parasites and provides the earliest yet identified event in the initiation of trypanosome differentiation.

## RESULTS

### TbPIP39 redistributes during synchronous differentiation.

**FIG 1:**
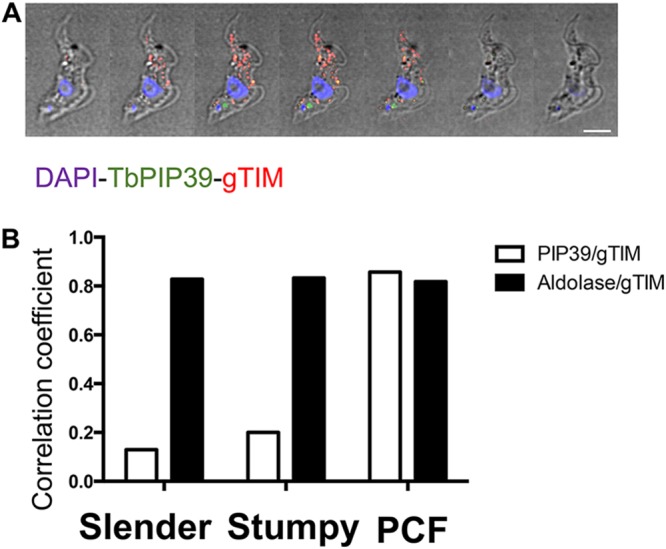
(A) Serial 0.3-μm Z stack slices through a stumpy-form trypanosome cell stained to localize the differentiation regulator TbPIP39 (green) or glycosomal TIM (red). The cell nucleus and kinetoplast are shown in blue. The TbPIP39 is located close to, but slightly anterior of, the kinetoplast and is not colocated with the glycosomal marker. DAPI, 4,6′-diamidino-2-phenylindole. Bar = 5 μm. (B) Pearson coefficient of colocalization between TbPIP39 and glycosomal TIM or between aldolase and glycosomal TIM in bloodstream slender and stumpy forms or in procyclic forms (PCF). Colocalization values were calculated using Volocity software based on captured confocal images. The threshold was set according to the background of images.

TbPIP39 is expressed in stumpy forms but not slender forms and is maintained in established procyclic forms in glycosomes (17). To assay the recruitment of TbPIP39 to glycosomes during differentiation, we analyzed parasites undergoing synchronous development from stumpy to procyclic forms. Specifically, a stumpy-enriched (80 to 90%) population was stimulated to undergo differentiation by exposure to 6 mM *cis*-aconitate *in vitro*. Interestingly, the stumpy-form location of TbPIP39 was distinct from that of the glycosomal marker protein glycosomal triosephosphate isomerase (gTIM). Rather than being detected in a punctate location throughout the cell typical of glycosomal staining, there was a concentration of staining close to the enlarged flagellar pocket, positioned between the nucleus and kinetoplast of the cell (Fig. 1A). In contrast, procyclic forms exhibited the expected distribution of TbPIP39, where colocalization with both glycosomal aldolase and gTIM was evident. To quantitate this location, slender-, stumpy-, and procyclic-form parasites were costained for TbPIP39 and gTIM or for aldolase and gTIM and analyzed by confocal microscopy, with colocalization determined by global Pearson correlation using Volocity software (www.perkinelmer.co.uk) (Fig. 1B). This confirmed that aldolase and gTIM showed a correlation of >80% in each life cycle stage, consistent with the ubiquitous expression of these marker glycosomal proteins. In contrast, the correlation of TbPIP39 and gTIM was less than 15% in slender forms (where TbPIP39 is not expressed at significant levels), and less than 20% in stumpy forms (where TbPIP39 is expressed), while in procyclic forms, the correlation was over 80%. This indicated that TbPIP39 may be recruited to glycosomes during the differentiation between bloodstream and procyclic forms.

**FIG 2:**
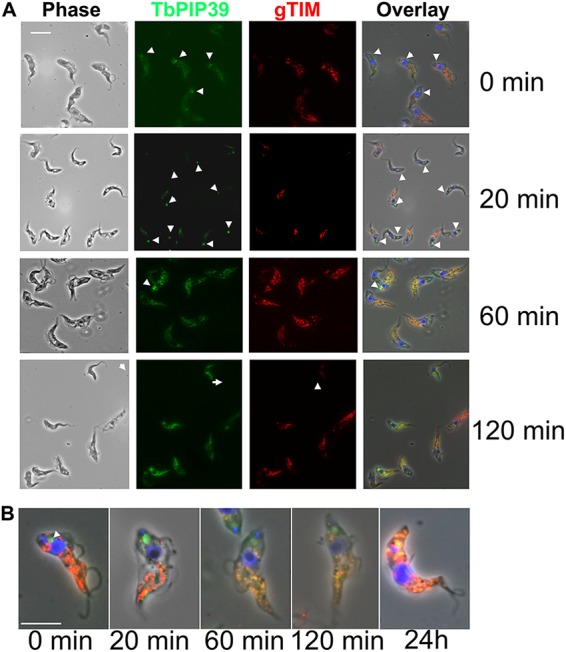
(A) Representative images of trypanosomes undergoing differentiation from stumpy forms to procyclic forms. Samples were taken at the time points indicated after the initiation of differentiation by *cis*-aconitate. Cells were labeled for the location of TbPIP39 (green) or the glycosomal marker gTIM (red), with the nucleus and kinetoplast being labeled with DAPI (4′,6-diamidino-2-phenylindole [blue]). Phase-contrast images are shown on the left-hand side, and merged images are shown on the right. Bar = 10 μm. (B) Selected fields of cells stained for TbPIP39, gTIM, and DAPI at time points after the initiation of differentiation. The location of TbPIP39 proximally to the flagellar pocket region of the cell is highlighted with arrowheads. Bar = 12 μm.

To analyze the kinetics of recruitment, the distributions of TbPIP39 and gTIM were analyzed at time points after exposure of stumpy-form parasites to 6 mM *cis*-aconitate *in vitro*. Figure 2A and B demonstrate that the periflagellar pocket staining of TbPIP39 was detectable at 0, 20, and 60 min following exposure to *cis*-aconitate, but was lost beyond that point. Conversely, a punctate glycosomal signal for TbPIP39 that colocalized with gTIM was detectable by 20 min, such that both a glycosomal location and periflagellar location were present for TbPIP39 at this early time point. At 60 min, the periflagellar staining was still detectable on some cells, but the glycosomal staining was more emphasized, and beyond this time point, the staining was mainly glycosomal.

**FIG 3:**
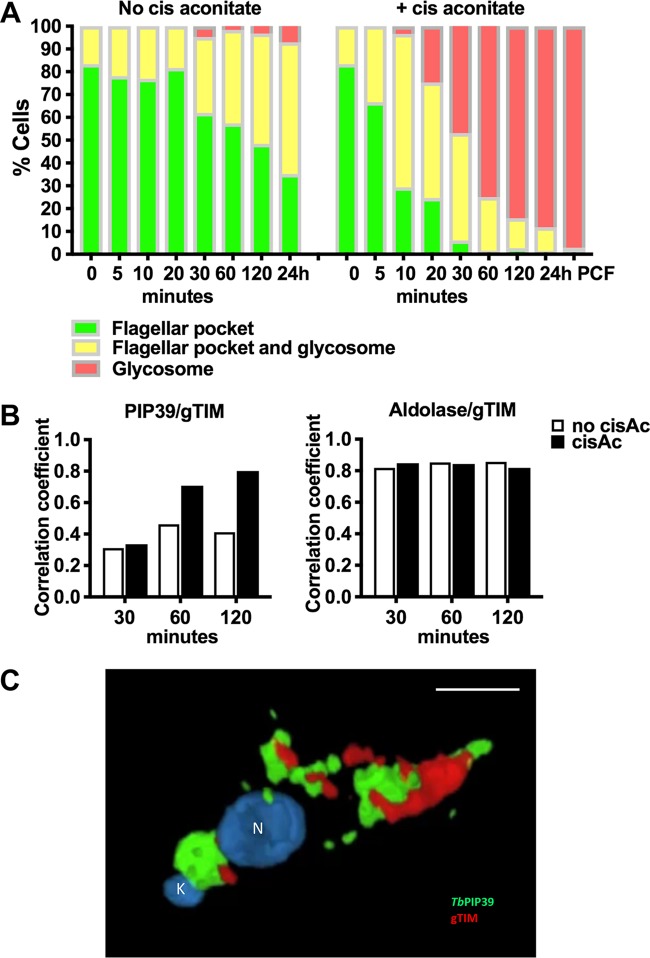
(A) Quantitation of the distribution of TbPIP39 between the periflagellar pocket location only (green), at the periflagellar pocket and in glycosomes (yellow), or exclusively in glycosomes (red). At each time point and under each condition (with or without *cis*-aconitate, to initiate differentiation), 250 cells were scored. In each case, cells remained in HMI-9 at 37°C to retain the viability of undifferentiated bloodstream-form parasites. (B) Pearson’s coefficient of colocalization between TbPIP39 and glycosomal TIM or between aldolase and glycosomal TIM at time points after the exposure of stumpy forms to *cis*-aconitate. Colocalization values were calculated using Volocity software based on captured confocal images. The threshold was set according to the background of images. *n* = 250 cells per condition/time point. (C) Three-dimensional reconstruction of a cell 30 min after exposure to *cis*-aconitate and stained for TbPIP39 (green) and glycosomal gTIM (red). The cell nucleus (N) and kinetoplast (K) are labeled blue. TbPIP39 is concentrated around the flagellar pocket of the cell but also shows labeling at a dispersed glycosomal location more anterior in the cell, coincident with the distribution of glycosomal gTIM. The labeling is exclusive so that merged staining associated with the coincident location of gTIM and TbPIP39 is not visible. Bar = 5 μm.

To quantitate the redistribution of the TbPIP39 signal, 250 cells were scored at each time point after exposure to *cis*-aconitate, and the parasites were assayed for signal either at the periflagellar pocket region alone, at the periflagellar pocket region and glycosomes, or in glycosomes alone. Figure 3A demonstrates that there was a transition during differentiation, with a predominantly glycosomal signal evident at 30 min and beyond in the differentiation time course. In contrast, parasites maintained *in vitro* at 27°C without *cis*-aconitate retained the signal close to the flagellar pocket, although there was also some glycosomal staining evident in some (20 to 50%) of the cells at 20 min and beyond. However, few (5%) cells exhibited only glycosomal staining, unlike when differentiation was stimulated with *cis*-aconitate, where >95% exhibited exclusively glycosomal location at 24 h. A global Pearson correlation analysis of parasites undergoing differentiation demonstrated that the colocalization of TbPIP39 and gTIM increased from around 30% at 30 min to nearly 80% at 120 min, highlighting the rapidity of the redistribution. In contrast, in the absence of *cis*-aconitate, the correlation never exceeded 40% (Fig. 3B). The correlation between aldolase and gTIM in the same analysis in contrast was consistently above 80%, demonstrating the stability of the colocalization between these glycosomal markers through the differentiation time course.

### Cytological analysis of the periflagellar staining of TbPIP39 in stumpy and differentiating cells.

**FIG 4:**
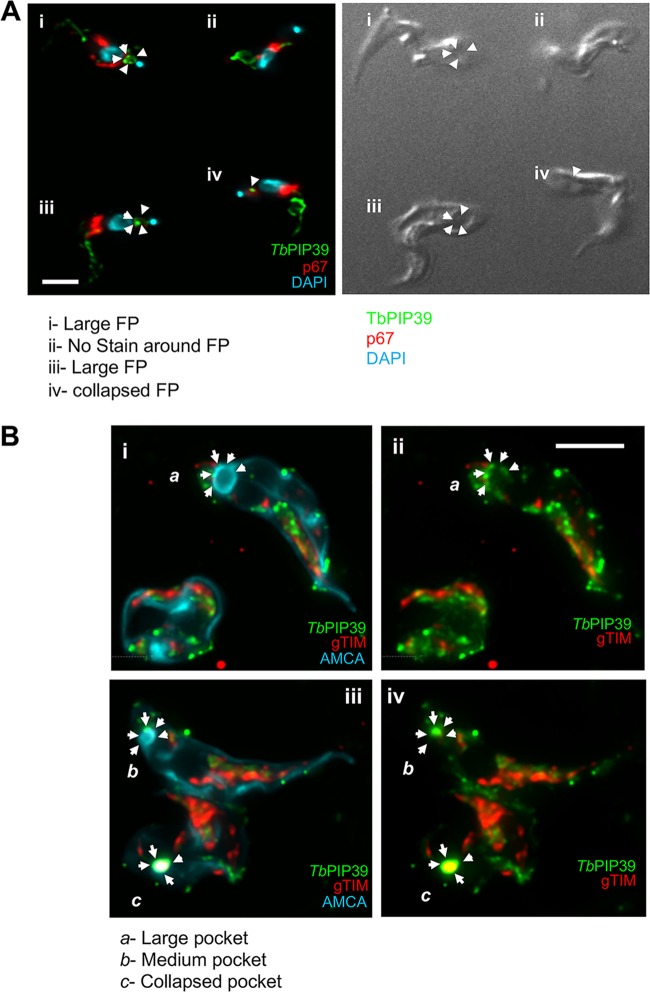
(A) Colabeling of stumpy-form cells (without exposure to *cis*-aconitate) stained for TbPIP39 (green) and the lysosomal marker p67 (red). The nucleus and kinetoplast are stained in blue. The TbPIP39 labeling is distinct from the lysosome, being positioned unevenly around the flagellar pocket (FP [cell i]) or at a tight focus in cells where the flagellar pocket is collapsed (cells iii and iv). Cell ii has no staining detected at the flagellar pocket region. Bar = 5 μm. (B) Colabeling of stumpy-form cells (without exposure to *cis*-aconitate) stained for TBPIP39 (green) and the glycosomal gTIM (red). The flagellar pocket and cell surface membrane are labeled blue with aminomethylcoumarin (AMCA). Arrows indicate the distribution of the TbPIP39 signal unevenly around the flagellar pocket (Cells *a* and *b* [images i and ii]), or at a tight focus associated with a collapsed flagellar pocket (cell *c* [images iii and iv]). Bar = 5 μm.

**FIG 5:**
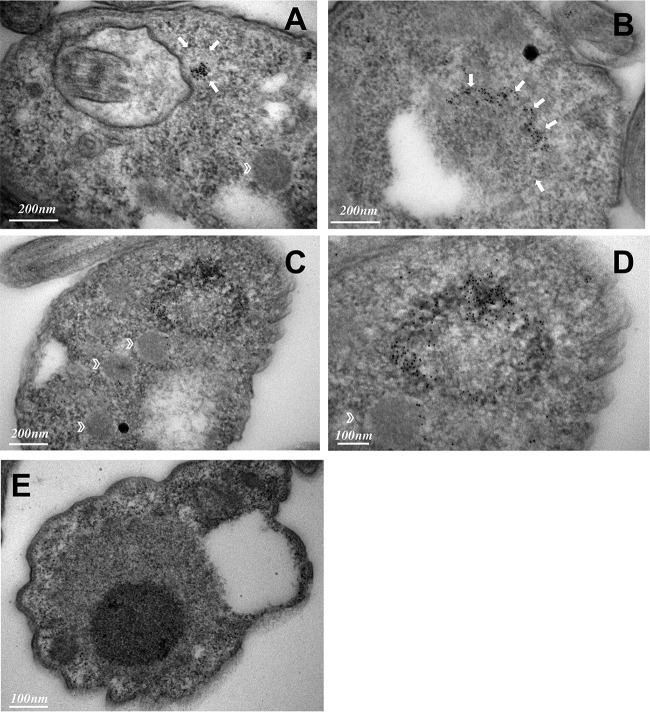
**(**A to D) Immunoelectron micrographs taken of thin sections of the flagellar pocket region of stumpy cells immunogold labeled for TbPIP39. Arrows indicate the boundary of membrane-bound vesicles containing TbPIP39 signal; glycosomes are indicated by chevrons in panels A, C, and D. (E) Immunoelectron micrographs of the flagellar pocket region of stumpy cells in the absence of primary antibody.

To examine the unusual periflagellar pocket location of TbPIP39 in stumpy forms more closely, we carried out confocal microscopy to visualize the three-dimensional distribution of TbPIP39 at 30 min after exposure to *cis*-aconitate. Figure 3C and Movie S1 in the supplemental material show the concentration of the TbPIP39 around the flagellar pocket of the cell. As expected, at 30 min after exposure to *cis*-aconitate, TbPIP39 was also detected in the glycosomal material anterior of the nucleus. Cells were also examined by deconvolution fluorescence microscopy after labeling for TbPIP39 and the lysosomal marker p67 (Fig. 4A). This again demonstrated the periflagellar pocket location of TbPIP39 but revealed that the signal was not evenly distributed around the flagellar pocket periphery but rather was concentrated at discrete foci around the pocket. Furthermore, in cells where the flagellar pocket was collapsed during fixation, the TbPIP39 focused to a tight point supporting the distribution of the signal around, not in, the flagellar pocket (Fig. 4B). The site of TbPIP39 localization was also investigated by immunoelectron microscopy (Fig. 5A to E). Although signal was not abundant, we were able to detect clusters of TbPIP39 labeling within the lumen of membrane-bound structures and at vesicle membranes distinct from the flagellar pocket membrane and close to glycosomes in this region of the cell.

10.1128/mBio.00875-19.6 MOVIE S1 Three-dimensional reconstruction of a cell 30 min after exposure to *cis*-aconitate and stained for TbPIP39 (green) and glycosomal gTIM (red). The cell nucleus (N) and kinetoplast (K) are labeled blue. TbPIP39 is concentrated around the flagellar pocket of the cell but also shows labeling at a dispersed glycosomal location more anterior in the cell, coincident with the distribution of glycosomal gTIM. Download Movie S1, MOV file, 0.2 MB. Copyright © 2019 Szöőr et al. 2019 Szöőr et al. This is an open-access article distributed under the terms of the Creative Commons Attribution 4.0 International license.

Finally, to evaluate whether the periflagellar pocket location of TbPIP39 was an artifact of indirect immunofluorescence, we generated cell lines with TbPIP39 fused to mNeonGreen (see Fig. S1 in the supplemental material) and used live-cell imaging to detect the fluorescent protein in stumpy forms restrained in a hydrogel matrix via video microscopy. Figure S1 and Movie S2A in the supplemental material reveal that in stumpy forms, the fluorescent protein localized at the same periflagellar pocket site, as previously visualized by immunofluorescence microscopy, a profile also seen with a yellow fluorescent protein (YFP)-TbPIP39 fusion protein and labeling using TbPIP39-specific antibody (not shown). Moreover, the signal redistributed to glycosomes in parasites exposed to *cis*-aconitate for 2 h (Movie S2B) and 24 h (Movie S2C).

10.1128/mBio.00875-19.2 FIG S1 (A) Schematic representation of the construct used to N-terminally tag TbPIP39 with mNeonGreen. (B) Captured stills from video images of TbPIP39 mNeonGreen signal in stumpy forms at 0 h or 2 or 24 h after exposure to *cis*-aconitate to initiate differentiation. These correspond to Movies S2A, B, and C. At 0 h, the signal is predominantly periflagellar pocket, though with some dispersed glycosomal signal. At 2 h, there is still some periflagellar pocket signal but stronger glycosomal signal. At 24 h, the signal is exclusively glycosomal. Bar = 20 μm. Download FIG S1, TIF file, 3.0 MB. Copyright © 2019 Szöőr et al. 2019 Szöőr et al. This is an open-access article distributed under the terms of the Creative Commons Attribution 4.0 International license.

10.1128/mBio.00875-19.7 MOVIE S2A Video images of TbPIP39 mNeonGreen signal in stumpy forms 0 h after exposure to *cis*-aconitate to initiate differentiation. At 0 h, the signal is predominantly periflagellar pocket, though with some dispersed glycosomal signal. Bar = 20 μm. Download Movie S2a, MOV file, 0.2 MB. Copyright © 2019 Szöőr et al. 2019 Szöőr et al. This is an open-access article distributed under the terms of the Creative Commons Attribution 4.0 International license.

10.1128/mBio.00875-19.8 MOVIE S2B Video images of TbPIP39 mNeonGreen signal in stumpy forms 2 h after exposure to *cis*-aconitate to initiate differentiation. Download Movie S2b, MOV file, 0.2 MB. Copyright © 2019 Szöőr et al. 2019 Szöőr et al. This is an open-access article distributed under the terms of the Creative Commons Attribution 4.0 International license.

10.1128/mBio.00875-19.9 MOVIE S2C Video images of TbPIP39 mNeonGreen signal in stumpy forms 24 h after exposure to *cis*-aconitate to initiate differentiation. Download Movie S2c, MOV file, 0.2 MB. Copyright © 2019 Szöőr et al. 2019 Szöőr et al. This is an open-access article distributed under the terms of the Creative Commons Attribution 4.0 International license.

Combined, our results demonstrated that TbPIP39 was positioned close to, but not in, the flagellar pocket of the stumpy form parasites and distributed to glycosomes within 1 to 2 h of the initiation of their differentiation to procyclic forms.

### Glycosomal dynamics during differentiation upon TbPIP39 depletion.

**FIG 6:**
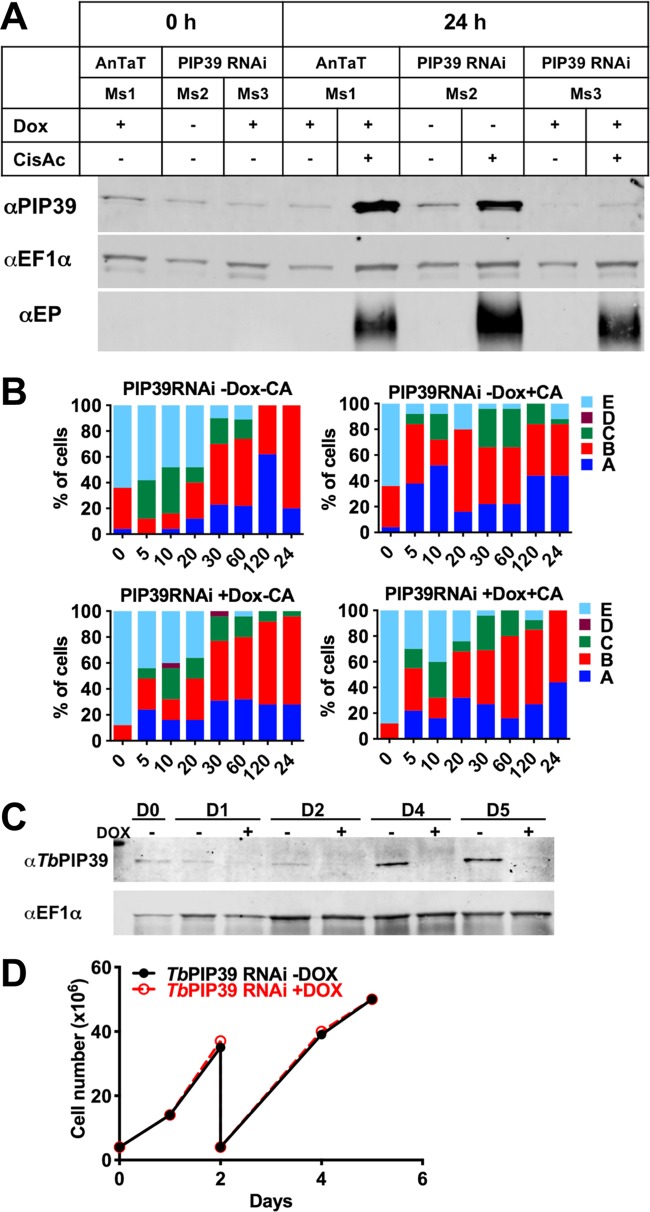
(A) RNAi depletion of TbPIP39. T. brucei EATRO 1125 AnTat1.1 90:13 TbPIP39 RNAi cells or T. brucei EATRO 1125 AnTat1.1 90:13 cells were grown in mice, with or without doxycycline induction. The generated stumpy cells were then exposed, or not, to *cis*-aconitate to initiate differentiation (with doxycycline remaining in the RNAi-induced samples), and TbPIP39 protein was detected at 24 h. TbPIP39 levels increase during differentiation, but this is greatly reduced in induced RNAi samples. EF1α provides a loading control, this being a little higher in cells exposed to *cis*-aconitate due to their replication as differentiated procyclic forms. EP procyclin staining shows relative differentiation in the presence or absence of *cis*-aconitate. (B) Distribution of the glycosomal marker aldolase and the lysosomal marker p67 during differentiation between stumpy and procyclic forms. The panel shows the prevalence of different categories of lysosomal/glycosomal staining (as defined by Herman et al. [23]) at times through differentiation when TbPIP39 was depleted by RNAi or not. The cytological profiles were as follows: A, enlarged lysosomal signal; B, lysosome enlarged but separated into distinct smaller vesicles; C, normal lysosomal size, with glycosomal colocalization; D, no lysosome observed; E, lysosome normal, with no colocalization with glycosomes. (C) Western blot of TbPIP39 in cells differentiated to proliferative procyclic forms with TbPIP39 RNAi induced or not. EF1α shows the loading control. The lower panel shows that by days 4 and 5, the cells remain proliferative (the cells were passaged at day 2) despite expressing significantly less TbPIP39.

During differentiation between stumpy forms and procyclic forms, there is turnover of the glycosomal population, presumably contributing to the metabolic adaptation of the parasites as they enter the tsetse fly. To determine whether TbPIP39 recruitment contributed to the control of glycosomal turnover and maturation during differentiation, we analyzed the distribution of glycosomal aldolase and the lysosomal marker p67 during differentiation with TbPIP39 either depleted or not by RNA interference (RNAi). Previously, lysosomal and glycosomal staining patterns during differentiation have been subjectively categorized according to the distribution of aldolase and p67 in several cytological subtypes (A to E) (23) (detailed in Fig. 6B). To determine if glycosomal/lysosomal dynamics were perturbed by reduced TbPIP39 recruitment during differentiation, pleomorphic T. brucei EATRO 1125 AnTat1.1. 90:13 bloodstream-form parasites—competent for stumpy formation and inducible RNA interference—were generated able to deplete TbPIP39 under doxycycline regulation. When grown in mice provided either with or without doxycycline in their drinking water, uniform populations of stumpy forms were generated whether TbPIP39 mRNA was targeted for RNAi or not. In mice, the low levels of TbPIP39 were not significantly further reduced by RNAi and normal differentiation to stumpy forms occurred, as previously seen (18). However, when these stumpy-form populations were induced to differentiate to procyclic forms with *cis*-aconitate, the normal increase of TbPIP39 levels was not observed over 24 h, supporting RNAi-mediated depletion during differentiation (Fig. 6A). Over this period, procyclin expression occurred but was less efficient than in uninduced parasites, highlighting delayed or reduced differentiation with a reduction in TbPIP39, as previously reported (18).

To determine the consequences of TbPIP39 RNAi for the glycosomal and lysosomal configurations, cells were assayed during differentiation for the abundance of each glycosomal/lysosomal category (A to E) subjectively defined by Herman et al. (23) (Fig. 6B). We observed no clear change in the distribution of the glycosome and lysosomal signal in either the presence or absence of *cis*-aconitate or when TbPIP39 was depleted or not. The differentiated cells were also allowed to proliferate as procyclic forms for 5 days after the initiation of differentiation with TbPIP39 RNAi depletion maintained or not with doxycycline. Figure 6C demonstrates that although TbPIP39 remained significantly reduced with RNAi induction in the differentiated procyclic cells, these grew at a level equivalent to that of cells where TbPIP39 was not depleted.

We conclude that preventing the accumulation of TbPIP39 by RNAi does not significantly alter the changes in the glycosomal dynamics during differentiation from stumpy forms to procyclic forms. Moreover, differentiated procyclic forms do not require abundant TbPIP39 to sustain *in vitro* procyclic-form growth. Instead the dominant function of TbPIP39 under the growth conditions used appears to be restricted to regulating the efficiency of differentiation between bloodstream and procyclic forms.

### TbPTP1 and REG9.1 localize close to TbPIP39 in stumpy forms.

**FIG 7:**
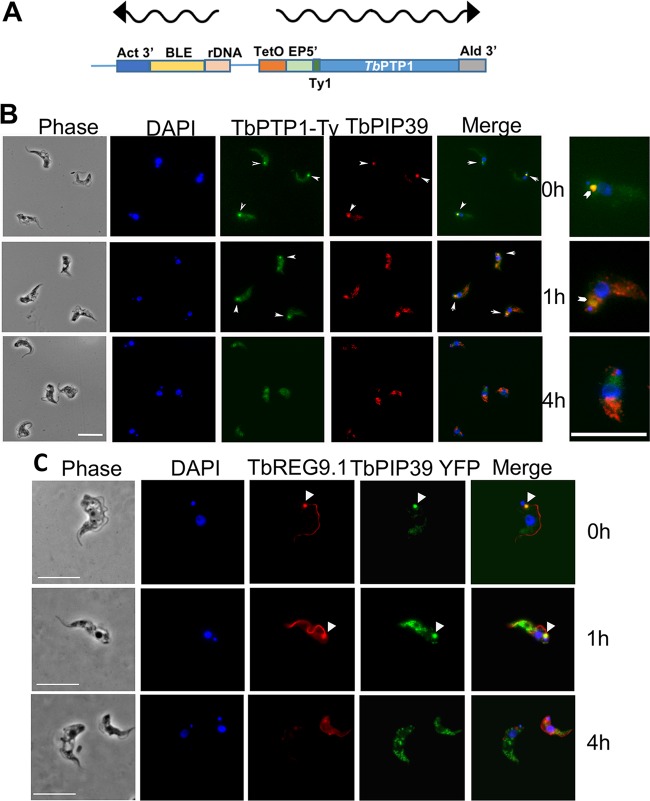
(A) Schematic representation of the construct used to N-terminally tag TbPTP1 with the Ty1 epitope tag. (B) Colocalization of TbPTP1-Ty1 (green), and TbPIP39 (red) in stumpy forms (0 h) and after 1 and 4 h of exposure to *cis*-aconitate. The TbPTP1 signal is detected as an ectopically expressed copy incorporating an N-terminal Ty1 epitope tag to allow its detection. The TbPIP39 signal is detected using an antibody recognizing that protein. The cell nucleus and kinetoplast are labeled with DAPI (blue). TbPTP1 colocalized with TbPIP39 in stumpy forms (arrowheads), but after 1 h, TbPIP39 has relocated to glycosomes, while TbPTP1 has become more diffuse, albeit some remains concentrated in the periflagellar pocket region (arrowheads). At 4 h, TbPIP39 is glycosomal, while TbPTP1 is diffuse throughout the cell body. Bar = 10 μm. Enlarged images on the right show selected cells from the merged panel to highlight signal at the periflagellar pocket region (chevron). (C) Detection of YFP-tagged TbPIP39 (green) in stumpy-form cells and its location with respect to REG9.1 (red). DAPI (blue) denotes the position of the cell nucleus and kinetoplast. Bar = 15 μm.

TbPIP39 is negatively regulated by the tyrosine phosphatase TbPTP1, which acts as an inhibitor of differentiation in stumpy forms. Previously, TbPTP1 had been difficult to localize using antibody specific for that molecule (16), and so we clarified its localization with respect to TbPIP39 using an inducible N-terminally Ty1 epitope-tagged copy (Fig. 7A). Here, TbPTP1 was detected at the discrete periflagellar pocket location, with TbPIP39 colabeling confirming that the location of the two signaling phosphatases was coincident in stumpy forms (Fig. 7B). After 1 h of exposure to *cis*-aconitate, TbPIP39 relocated to glycosomes, as seen earlier, whereas TbPTP1 became more diffuse at the periflagellar pocket site, and by 4 h, the signal was distributed throughout the cell body in the differentiating cells. Thus, TbPTP1 and TbPIP39 are at the same cellular site when the stumpy forms receive the signal to differentiate.

The TbPIP39/TbPTP1 node was also similar to the location of REG9.1, a regulator of stumpy-specific transcripts previously observed at the periflagellar pocket region of stumpy forms, in addition to along the flagellum or flagellum attachment zone (FAZ) (24). Therefore, we colocalized REG9.1 with epitope-tagged TbPIP39 using a REG9.1-specific antibody during differentiation and observed redistribution from the periflagellar pocket node to the cytoplasmic distribution previously in stumpy forms within 4 h. Hence, TbPIP39, TbPTP1, and REG9.1 are all colocalized in stumpy forms before their redistribution and separation at the onset of differentiation.

### The periflagellar location of TbPIP39 and TbPTP1 coincides with flagellar pocket ER.

**FIG 8:**
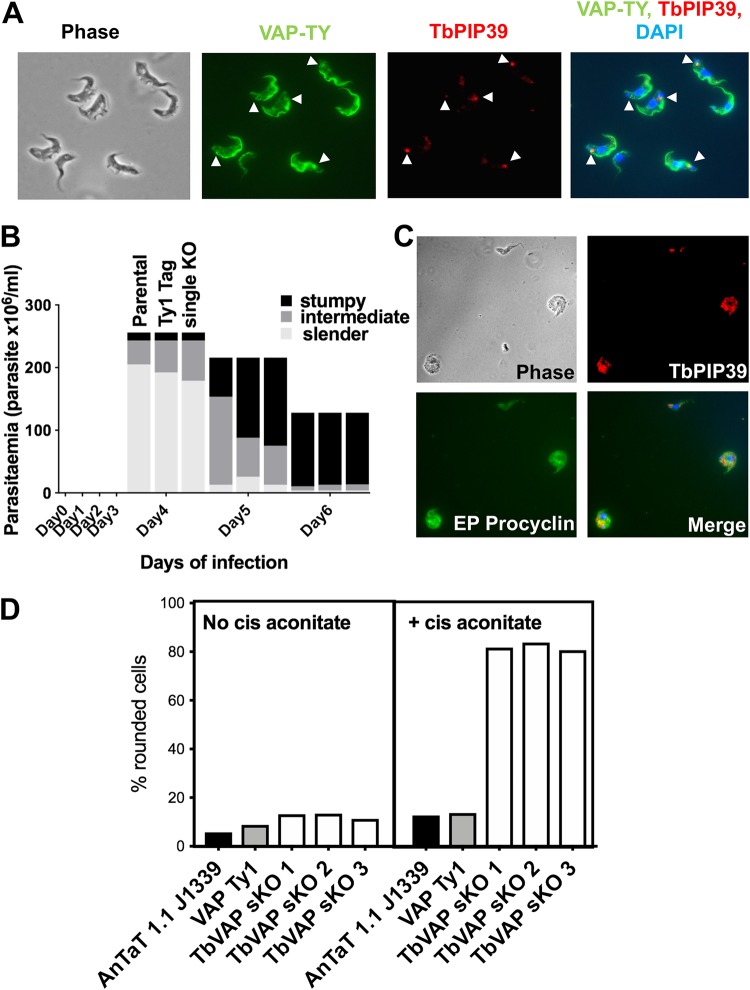
(A) Detection of Ty1 epitope-tagged TbVAP in stumpy-form cells and its location with respect to TbPIP39. The Ty1-tagged TbVAP (green) is detected at the flagellar pocket region, along the flagellum attachment zone, and in the cell body. TbPIP39 (red) is closely proximal at the flagellar pocket region but not precisely coincident. DAPI (blue) denotes the position of the cell nucleus and kinetoplast. Arrowheads indicate the region of TbPIP39. Bar = 15 μm. (B) Proportion of slender, intermediate, and stumpy forms in TbVAP single-knockout cells versus parental cells or epitope-tagged TbVAP cells. (C) TbVAP single-allele-replacement mutants 24 h after the initiation of differentiation to procyclic forms. The differentiating cells (indicated by their expression of EP procyclin [green]) are swollen and lose integrity. TbPIP39 is punctate throughout the cell, indicating a glycosomal rather than periflagellar pocket distribution. The merged panel shows TbPIP39 (red), EP procyclin (green), and DAPI (blue) revealing the nucleus and kinetoplast. Bar = 25 μm. (D) Quantitation of cells that exhibit a rounded morphology. Parental T. brucei AnTat1.1 J1339 cells, TbVAP1-mNeonGreen-Ty1-expressing cells, or three TbVAP single-allele-deletion mutants (sKO) are shown. In each case, the percentage of rounded cells is shown after 24 h in either the absence or presence of *cis*-aconitate as a differentiation stimulus. At least 250 cells were scored for each cell line and each condition.

The observed location of TbPIP39 in stumpy forms was reminiscent of a specialized region of the ER at the flagellar pocket identified by electron tomography of procyclic-form cells (22). This region of the cell is defined by the presence of TbVAP, a flagellum attachment zone (FAZ) ER membrane contact protein. TbVAP depletion by RNAi in procyclic forms results in loss of the FAZ ER and flagellar pocket ER, but no detectable growth deficit (22). To determine if the TbPIP39 location in stumpy forms associated at the flagellar pocket ER, we colocalized TbPIP39 with TbVAP detected by its incorporation of N-terminal mNEONGreen Ty1 epitope tag and expression in pleomorphic cells. Figure 8A demonstrates that the TbPIP39 and TbVAP signals are closely located in the flagellar pocket region of stumpy forms, but not completely coincident, with TbPIP39 sometimes between two separated TbVAP foci. Matching previous analysis in procyclic-form cells (22), TbVAP signal also extended along the flagellum attachment zone with further staining in the cell body.

To explore the association of TbPIP39 and TbVAP at this periflagellar pocket location in more detail, we exploited CRISPR (clustered regularly interspaced short palindromic repeats) to delete the TbVAP gene in pleomorphic bloodstream-form trypanosomes (25). Thus, the Cas9-expressing cell line T. brucei EATRO 1125 AnTat90:13 J1339 (26) was transfected with repair templates targeted to the TbVAP gene genomic location, and mutants were isolated (see Fig. S2A in the supplemental material). The resulting cell lines were found to retain a TbVAP gene copy despite their successful insertion of the drug resistance gene (Fig. S2B to D); further attempts to delete the remaining allele were unsuccessful. These TbVAP-depleted cells exhibited stumpy formation *in vivo* with similar kinetics to wild-type cells (Fig. 8B), allowing the location of TbPIP39 and its relocation after the initiation of differentiation to procyclic forms to be assayed.

10.1128/mBio.00875-19.3 FIG S2 (A) Schematic representation of the deletion of TbVAP alleles by CRISPR. The allele was deleted through integration of a blasticidin resistance cassette. (B) PCR amplification to monitor TbVAP replacement of 4 cell lines isolated after transfection with the blasticidin replacement cassette. The TbVAP gene was not detected except in the parental line (using primers TbVAP and TbVAP 5′ untranslated region [UTR]), whereas integration of the TbVAP replacement cassette was seen (detected with primers CRISPR 1 and 7). However, after growth *in vivo*, the TbVAP gene was detected after differentiation to stumpy forms, despite not being detected in slender forms infected with the same cell lines. In each case, primers detecting the puromycin resistance gene confirmed amplifiable DNA was present in each sample. Potentially, this indicated that cells retaining TbVAP had been selected during growth in vivo from a mixed population of TbVAP null and single-allele-replacement cells. (C) In case a mixed population of null mutants and single-allele-replacement lines had been isolated, cell lines B1 and C5 were cloned, generating lines B1 (C9, C11, D3, D5, and D7) and C5 (F2, F4, G4, and G9). Analysis of these clones demonstrated that all had inserted the TbVAP replacement cassette (detected using primers CRISPR 1 and 7). The Purobox primers confirmed the presence of equivalent amounts of amplifiable DNA from each line. Par, parental line. (D) PCR analysis of the presence of TbVAP gene in the transfected and cloned lines. All cell lines were found to retain an intact copy of the TbVAP gene, detected using primers TbVAP and TbVAP 5′ UTR. Retransfection of these single-allele-replacement lines with a hygromycin TbVAP replacement cassette did not result in the isolation of viable transfectants, suggesting TbVAP may be essential. Download FIG S2, TIF file, 2.4 MB. Copyright © 2019 Szöőr et al. 2019 Szöőr et al. This is an open-access article distributed under the terms of the Creative Commons Attribution 4.0 International license.

In TbVAP single-knockout (single-KO) stumpy forms, TbPIP39 was detected at the periflagellar pocket location, similar to that seen in wild-type parasites. Correspondingly, when cells were induced to differentiate to procyclic forms with *cis*-aconitate, TbPIP39 relocated to glycosomes and the cells expressed procyclin, indicating that TbVAP depletion did not alter glycosomal TbPIP39 loading or differentiation (not shown). However, when the TbVAP single-KO cells were analyzed after 24 h under differentiation conditions (i.e., with *cis*-aconitate), the parasites appeared enlarged and rounded and were dying, unlike wild-type parasites at this time point (Fig. 8C) (mean = 82% from an analysis of three independent null mutant lines, versus 13% and 14% in the parental and VAP-Ty1 lines, respectively; *n* = 250 cells per sample [Fig. 8D]). Thus, both alleles of TbVAP are necessary for the viability of differentiating cells, but not for the normal relocation of TbPIP39 early in the process.

## DISCUSSION

The differentiation of African trypanosomes between life cycle stages is enacted rapidly upon transition from the blood of mammalian hosts to the midgut of the tsetse fly. We have shown previously that two phosphatases are important in the signaling of the changes from one environment to the next: TbPTP1 and TbPIP39. Of these, TbPIP39 is glycosomal in procyclic forms, signaled through its C-terminal peroxisomal targeting signal 1 (PTS 1). Here we show that when first made in stumpy forms, TbPIP39 is not glycosomal but rather is localized at a periflagellar pocket region of the parasite, where it colocalizes with the differentiation inhibitor TbPTP1. However, upon reception of the differentiation signal, TbPIP39 is rapidly (within approximately 20 min) relocated into glycosomes, whereas TbPTP1 becomes dispersed to a nonglycosomal, possibly cytosolic site. It is also coincident with the location of a regulator of stumpy-form transcripts, REG9.1. Interestingly, this periflagellar pocket site is close to the specialized FAZ endoplasmic reticulum, defined by TbVAP, which may represent a site of glycosomal biogenesis in the differentiating cells. We propose this molecular node comprising TbPIP39, TbPTP1, and REG9.1 generates a “stumpy regulatory nexus” (STuRN) where the events initiating differentiation between bloodstream and procyclic forms occur.

The biogenesis of peroxisomes and glycosomes can involve new organelles that arise from preexisting peroxisomal/glycosomal structures or *de novo* synthesis from the endoplasmic reticulum. However, in eukaryotes subject to environmental change, peroxisome composition can be modulated to allow metabolic adaptation, and this can be achieved by *de novo* loading from the ER (27) as well as the growth and division of existing glycosomes. The dynamics of environmental adaptation in procyclic-form parasites have been analyzed both during differentiation and upon exposure of procyclic forms to low and high glucose concentrations. In differentiation, autophagy is considered important due to the coassociation of lysosomes and glycosomes early in the transition between stumpy and procyclic forms (23). The assessment of this is relatively subjective, and we observed limited coassociation between the lysosomal marker p67 and glycosomal aldolase during synchronous differentiation—at least in the first 2 h. In contrast, TbPIP39 relocated from the STuRN to glycosomes within 20 min, this region being neither the flagellar pocket lumen nor the flagellar pocket membrane. Instead the STuRN was adjacent to a specialized region of the endoplasmic reticulum that has been visualized by electron tomography of procyclic-form cells (22) and is also the site of ER concentration in procyclic forms in low glucose. In this region of the cell, ER is associated with both the flagellar pocket and flagellum attachment zone, the region being defined by TbVAP, an orthologue of VAMP-associated protein. This molecule is proposed to coordinate ER in this region of the cell through linking of the specialized four microtubules positioned at flagellar pocket region of the cytoskeleton to the endoplasmic reticulum or controlling interaction between central ER and the flagellar pocket-associated ER. In a previous study, procyclic-form viability was not compromised by efficient RNA interference targeting TbVAP, suggesting that it is not essential or that significant depletion of its protein levels after RNA interference does not compromise cell viability and replication. By CRISPR/Cas9-mediated gene deletion, we also found that bloodstream forms were viable after the deletion of one allele, although both alleles could not be deleted. This may reflect that the protein is essential, contrasting with RNAi depletion experiments, although technical reasons cannot be excluded. Interestingly; however, the TbVAP single-KO mutants—although able to initiate differentiation between stumpy and procyclic forms—lost cell integrity after 24 h and appeared swollen and balloon like. Although this suggests that the levels of this molecule are important during differentiation, we have not been able to assay TbVAP levels due to the absence of an antibody detecting the protein.

**FIG 9:**
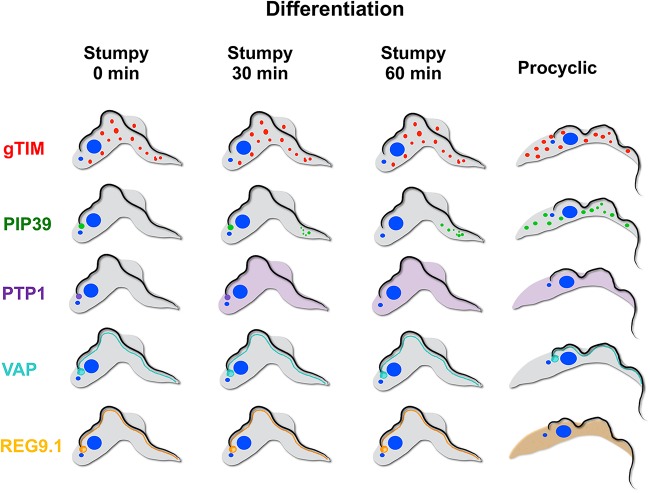
Schematic representation of the distribution of gTIM (representing glycosomes) TbPIP39, TbPTP1, TbVAP, and REG9.1 in stumpy forms and at 30 and 60 min after exposure to *cis*-aconitate and in procyclic forms.

Our data invoke a model where in stumpy forms, TbPIP39 is poised for glycosome recruitment through its recruitment to the STuRN in a preglycosomal concentration similar to preperoxisomal vesicles (Fig. 9). At this site, the presence of TbPTP1 inhibits its activity. With the initiation of differentiation, however, TbPTP1 is inactivated and the TbPIP39 protein is activated by phosphorylation through the activity of an as-yet-unidentified kinase and assembled into glycosomes, where it can no longer be accessed by TbPTP1. Removing the inhibitor TbPTP1 from its substrate, TbPIP39, renders the differentiation signaling irreversible—a commitment event that has been mapped to approximately 1 h after exposure to citrate/*cis*-aconitate (18, 28), coincident with the dispersal of TbPIP39 and TbPTP1 in differentiating cells. This approximates to the timing of the commitment to differentiation, but the basis of repositioning of the TbPIP39 is unknown: the protein has a glycosomal targeting signal, but we have not detected, for example, phosphorylation changes that might license glycosomal relocation after the initiation differentiation (28). It is also formally possible that TbPIP39 is not relocalized, but instead is lost from the periflagellar pocket site and replaced by new TbPIP39 recruited to glycosomes. In either scenario, the glycosomal pool rapidly turns over through autophagy during differentiation (23), and the biogenesis of new glycosomes, potentially generated at the flagellar pocket region, allows the remodeling of the glycosomal pool to its procyclic composition. It remains to be established whether TbPIP39 activity is necessary in the mature glycosomes that arise during differentiation or in procyclic forms, although the protein is abundant and retained in proliferative procyclic forms. Indeed, it will be interesting to explore the fitness of procyclic forms depleted of TbPIP39 under different culture conditions more closely mimicking conditions in the fly gut, such as when alternative carbon sources are available (29, 30).

The synchronous differentiation of trypanosome parasites and their developmental adaptation of glycosomal composition provide a unique capability to explore the dynamics of organellar development in an evolutionarily divergent eukaryotic model. This study provides the first temporal and positional tracking of signaling molecules, organellar compartments, and contact points at the STuRN, a potential site of glycosomal biogenesis/regeneration as the parasite initiates its metabolic adaptation to a nutritionally distinct environment. Coupled with the high-definition understanding of the structural organization and cytoskeletal interactions in this region of the highly ordered parasite cell (31–33), trypanosomes provide an invaluable model for the precise regulation and kinetics of interorganellar exchange in a eukaryotic cell.

## MATERIALS AND METHODS

### Parasites. (i) Cell lines and culturing *in vitro*.

Pleomorphic Trypanosoma brucei EATRO 1125 AnTat1.1 90:13 (TETR T7POL NEO HYG) (19) and EATRO AnTat 1.1 J1139 (26) parasites were used throughout.

Pleomorphic bloodstream and double-marker 29-13 procyclic-form trypanosomes (34) were cultured *in vitro* in HMI-9 (35) medium at 37°C in 5% CO_2_ or in SDM-79 (36) medium at 27°C, respectively.

The following selective drugs were used: hygromycin (2.5 μg/ml), puromycin (0.5 μg/ml), and blasticidin (2.5 μg/ml).

**(ii) *In vivo* studies.** Trypanosome infections were carried out in female healthy outbred MF1 mice at least 10 weeks old, immunocompromised with 25 mg/ml cyclophosphamide delivered intraperitoneally 24 h prior to trypanosome infection.

No blinding was performed, and the animals were not subject to previous procedures or drug treatment. Animal experiments were carried out according to the United Kingdom Animals (Scientific Procedures) Act under a license (PPL60/4373) issued by the United Kingdom Home Office and approved by the University of Edinburgh local ethics committee. Animals were kept in cages containing 1 to 5 mice on a 12-h daylight cycle and maintained at room temperature.

*In vivo* growth involved intraperitoneal injection of 10^5^ parasites into cyclophosphamide-treated mice, and the course of parasitemia was recorded by performing daily tail snips to estimate parasite numbers using a “rapid-matching” method involving visual comparisons of live parasites in blood by microscopy with a published standardized chart of parasite numbers per milliliter (37).

Ectopic gene expression was induced by inclusion of doxycycline (200 mg/ml in 5% sucrose) in the drinking water, with control mice being provided with 5% sucrose alone. Between 2 and 3 mice were used per group.

Stumpy-enriched populations were obtained 6 to 7 days after infection by DEAE cellulose purification (38).

For the initiation of differentiation, conditions were used as described in reference 17.

**(iii) Parasite transfection.** Parasite transfection was by Amaxa nucleofection according to previous detailed methods for pleomorphic (39) and 29-13 procyclic-form parasites (40).

### Plasmid construction and cell line generation. (i) Generating endogenously tagged TbPIP39 pleomorph cell lines.

Primers 1 to 4 were used to endogenously tag the N terminus of TbPIP39 using pPOTv4YFP and pPOTv6 mNEONGreen plasmids according to Dean et al. (41).

**(ii) Generating epitope-tagged TbPTP1 pleomorph cell lines.** The TbPTP1 open reading frame was amplified from T. brucei EATRO 1125 AnTat1.1 wild-type genomic DNA by PCR using primers 5 and 6 (see Table S1 in the supplemental material) with SpeI and BglII restriction sites for insertion into the pDex577-Y vector (42) for tetracycline-inducible overexpression with an N-terminal TY epitope tag. The resulting overexpression constructs were linearized with NotI and transfected into *Trypanosoma bucei* EATRO 1125 AnTat1.1 90:13 pleomorph cells. Several independent cell lines were isolated, and their growth was analyzed *in vitro* or *in vivo* in the presence or absence of tetracycline or doxycycline, respectively. Expression was confirmed by Western blotting using an anti-TY antibody.

10.1128/mBio.00875-19.4 TABLE S1 Oligonucleotides used in the study. Download Table S1, DOCX file, 0.01 MB. Copyright © 2019 Szöőr et al. 2019 Szöőr et al. This is an open-access article distributed under the terms of the Creative Commons Attribution 4.0 International license.

**(iii) Generating endogenously tagged and knockout TbVAP pleomorph cell lines.** The LeishGEdit program was used (25) to design oligonucleotide primers (Table S1, primers 7 to 11) to produce DNA fragments and single guide RNAs (sgRNAs) for the production of TY mNEONGreen-tagged VAP and KO VAP cell lines. To create the KO and endogenously tagged pleomorph cell lines, EATRO AnTat 1.1 J1139 cells were transfected as described in reference 26.

Several independent cell lines were isolated, and their growth was analyzed *in vitro* or *in vivo*. Pleomorph cell lines with the mNEONGreen-TY-tagged TbVAP were identified by Western blotting and immunofluorescence using an anti-TY antibody.

Several TbVAP knockout cell line candidates were isolated, and genomic DNAs were purified (QIAGene genomic DNA kit). The genomic DNAs were used in PCRs to confirm the presence of the blasticidin drug resistance cassette (replacing the endogenous TbVAP) (Table S1, primers 7 to 10) and also the lack of the endogenous TbVAP gene (Table S1, primers 14 and 15). TbPIP39 RNAi lines were described in reference 17.

### Western blotting, immunofluorescence, and confocal, immunoelectron microscopy and live-cell microscopy.

Protein expression analyses by Western blotting were carried out according to reference 17.

Approximately 1 × 10^9^ EATRO AnTat1.1 90:13 stumpy cells (around 90 to 95% cells of the isolated cells were stumpy forms, and the rest were intermediates) from mice were purified on DE52 column in PSG buffer (phosphate-buffered saline [PBS] plus glucose).

After purification, cells were resuspended in HMI9 at 4 × 10^6^/ml and left in a 37°C CO_2_ incubator for 60 min to recover. Then the culture was divided into two aliquots: ∼5 × 10^8^ stumpy cells were treated with *cis*-aconitate (+CA sample), and 5 × 10^8^ stumpy cells were left untreated (−CA sample). After 60 min of CA induction, cells were harvested by centrifugation, washed in 15 ml of Voorheis's modified phosphate-buffered saline (vPBS; PBS supplemented with 10 mM glucose and 46 mM sucrose, pH 7.6) and repelleted, before resuspension in 10 ml vPBS (2.5 × 10^8^ cells/10 ml). Immunofluorescence was carried out according to reference 20. Phase-contrast and immunofluorescence microscopy images were captured on a Zeiss Axioskop2 (Carl Zeiss microimaging) with a Prior Lumen 200 light source using a QImaging Retiga 2000R charge-coupled device (CCD) camera; the objective was a Plan Neofluar ×63 (1.25 NA). Images were captured via QImage (QImaging). Cells were captured for confocal microscopy or processed for immunoelectron or live-cell microscopy as described in Text S1 (43, 44) in the supplemental material.

10.1128/mBio.00875-19.1 TEXT S1 Supplemental materials and methods. Download Text S1, DOCX file, 0.02 MB. Copyright © 2019 Szöőr et al. 2019 Szöőr et al. This is an open-access article distributed under the terms of the Creative Commons Attribution 4.0 International license.

10.1128/mBio.00875-19.5 TABLE S2 Antibodies used in the study. Download Table S2, DOCX file, 0.01 MB. Copyright © 2019 Szöőr et al. 2019 Szöőr et al. This is an open-access article distributed under the terms of the Creative Commons Attribution 4.0 International license.
