# Supplementary figures and images for: Positional Dynamics and Glycosomal Recruitment of Developmental Regulators during Trypanosome Differentiation
Source: mBio. 2019 Jul 9;10(4):e00875-19. doi: 10.1128/mBio.00875-19 (PMC6747725; doi:10.1128/mBio.00875-19)

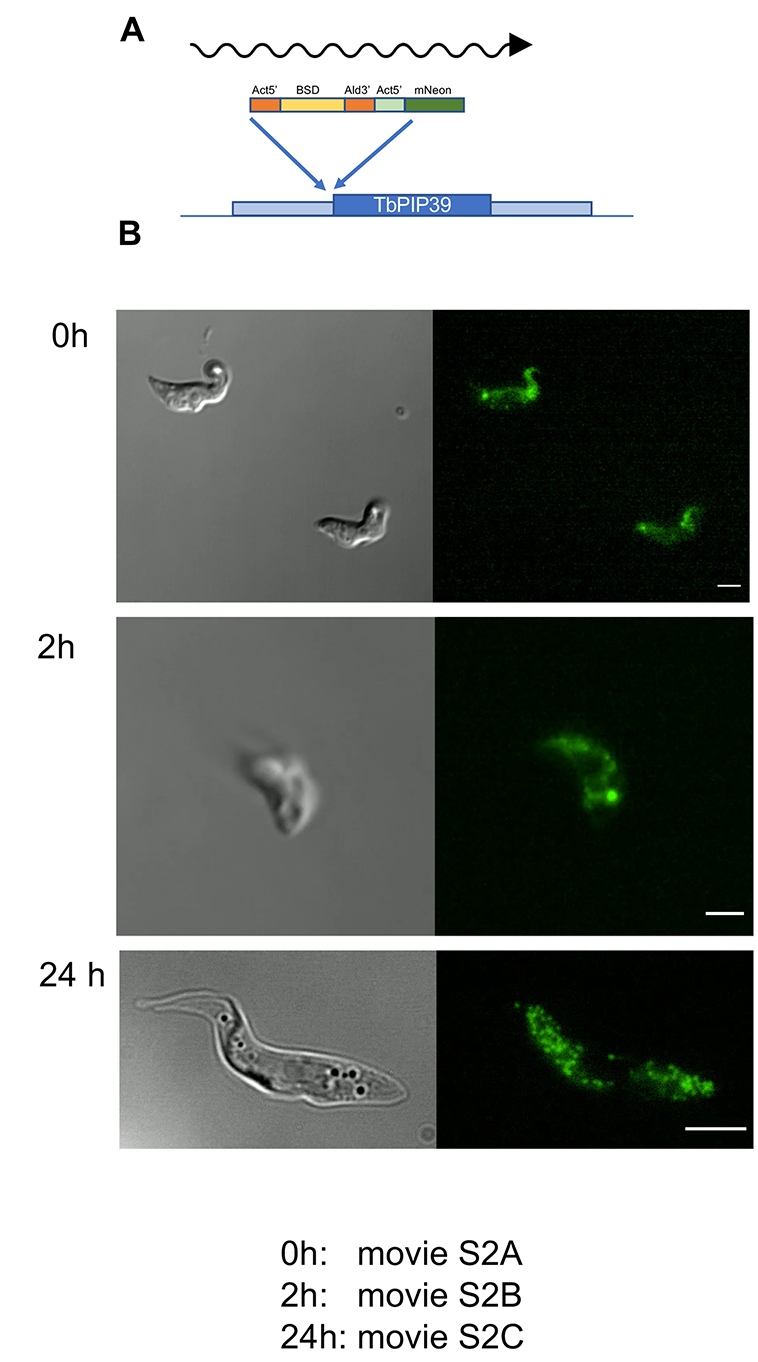

Supplement: FIG S1 [file mBio.00875-19-sf001.tif]

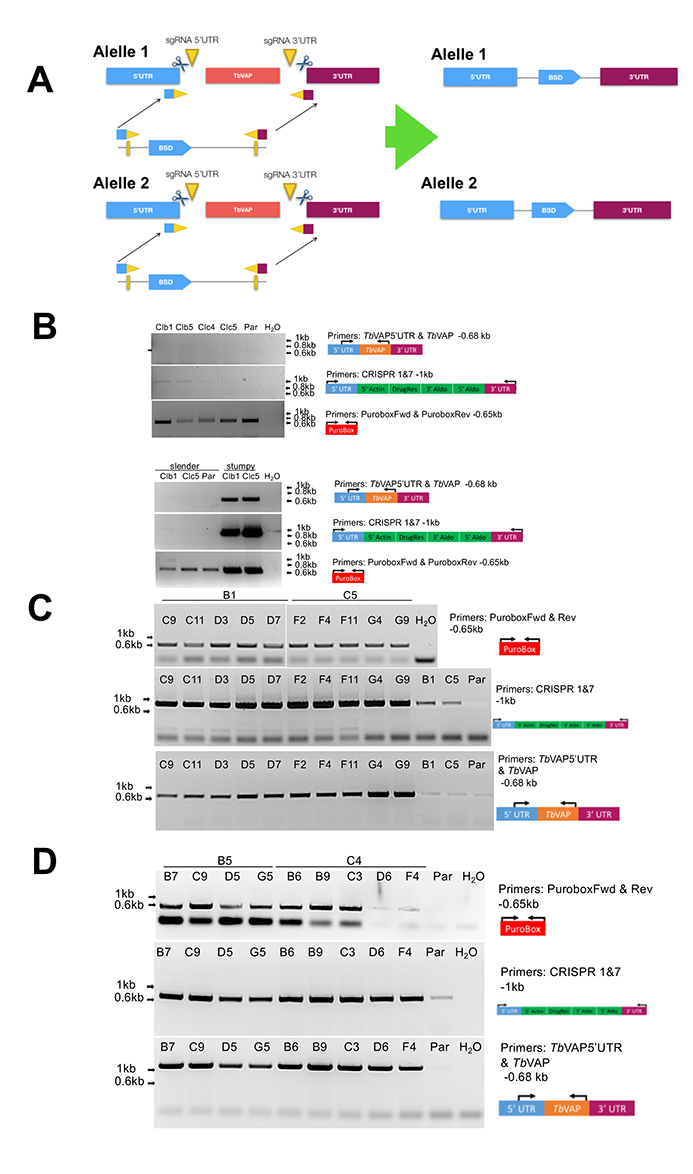

Supplement: FIG S2 [file mBio.00875-19-sf002.tif]
