# Supplementary material for: Positional Dynamics and Glycosomal Recruitment of Developmental Regulators during Trypanosome Differentiation
Source: mBio. 2019 Jul 9;10(4):e00875-19. doi: 10.1128/mBio.00875-19 (PMC6747725; doi:10.1128/mBio.00875-19)
Supplement: TABLE S1 [file mBio.00875-19-st001.docx]

**Table S1. Oligonucleotides used in the study**

| Number | Target gene | Primer name | Primer sequence |
| --- | --- | --- | --- |
| 1. | PIP39 | coding seq amplification Fwd | 5’ ggatcaggatcgggtagtatggtgaggacgacacgcttttc 3’ |
| 2. | PIP39 | coding seq amplification Rev | 5’ gagattccaccacgtgcctc 3’ |
| 3. | PIP39 | 5'UTR seq amplification Fwd | 5’ atgcgactcttggtctcacc 3’ |
| 4. | PIP39 | 5'UTR seq amplification Rev | 5’ gcagcaggtctgcattatacatttcaatgagttccagttggg 3’ |
| 5. | PTP1 | pDEX577SpeIFwd | 5’ ccactagtatgtccacagcgaagagttttccgatggctcaa 3’ |
| 6. | PTP1 | pDEX577BglIIRev | 5’ gggagatctcgcttttaagttaagtgtcgacaccagtcc 3’ |
| 7. | VAP | Upstream Fwd | 5’ acctgcacaaatatactctgaaagcaaccagtataatgcagacctgctgc 3’ |
| 8. | VAP | Upstream Rev | 5’ aacagacggtgccgccgttttacttctcatactacccgatcctgatccag 3’ |
| 9. | VAP | Downstream Fwd | 5’ acgttgttttgctggtggtggtttgcctacggttctggtagtggttccgg 3’ |
| 10. | VAP | Downstream Rev Tag | 5’ ttctgttcttttttccccccctcttttcctccaatttgagagacctgtgc 3’ |
| 11. | VAP | Downstream Rev KO | 5’ ttctgttcttttttccccccctcttttcctccggaaccactaccagaacc 3’ |
| 12. | VAP | Synthetic single guide RNA 5’ | 5’ gaaattaatacgactcactataggtatcttacaattttctgcgtgttttagagctagaaatagc 3’ |
| 13. | VAP | Synthetic single guide RNA 3’ | 5’ gaaattaatacgactcactataggagtgagtgtgctttcagaaagttttagagctagaaatagc 3’ |
| 14. | VAP | VAP Fwd | 5’ actctgaaagcaaccaacgc 3’ |
| 15. | VAP | VAP Rev | 5’ ccaccaccagcaaaacaacg 3’ |
