# Supplementary material for: Positional Dynamics and Glycosomal Recruitment of Developmental Regulators during Trypanosome Differentiation
Source: mBio. 2019 Jul 9;10(4):e00875-19. doi: 10.1128/mBio.00875-19 (PMC6747725; doi:10.1128/mBio.00875-19)
Supplement: TEXT S1 [file mBio.00875-19-s0001.docx]

***Text S1***

**Supplementary material and methods**

**Confocal imaging**

Confocal imaging used a Leica SP5 confocal laser scanning microscope, using 63x oil immersion objective (NA = 1.4) and 4.2x digital zoom. The green channel was imaged using a 488-nm argon laser, and the red channel was imaged using a 543-nm helium/neon laser. The final image was acquired using Volocity Software (Improvision Ltd.) version 4.4. 3D fluorescence microscopy was performed using a fully automated Leica DMI6000B inverse microscope equipped with a 100x (NA 1.4) oil immersion objective and a Leica DFC365 FX CCD camera. Fluorescent and differential interference contrast (DIC) image stacks were acquired by recording stacks of 50 images with a step size of 137 nm. Fluorescent image stacks were deconvolved using Huygens Essential (SVI, Hilversum, Netherlands). Images were visualised as z-stack projections using Fiji(43), or as volumetric representations using Amira (Thermo Fisher Scientific).

**Electron microscopy**

After incubation of cells in an equal volume of vPBS and 8% paraformaldehyde, cells were pelleted at 1,000x g for 15 min at 4^o^C. The supernatant was removed and the pellet dehydrated with 30% ethanol at 4^o^C for 30min. The pellet was then taken through the following series of methanol dehydrations, 60%, 90%, 3 x absolute (30 min each) at -20^o^C. After dehydration, the pellet was incubated in 2mL of 30% HM20 Lowicryl monostep EM resin (EMS - 14345) in methanol at -20^o^C, 30min, then taken through increasing concentrations of HM20 (60 min each) then 3 x 100% absolute HM20 at -20^o^C. The pellet was polymerised at -20^o^C for 24hr using UV light at 360nm, then left at -20 ^o^C for 24h. The block was cut using a diamond knife and 100nm thin sections were incubated on 100µL droplets of 100mM glycine in PBS 2 x 10 min. Sections were then probed with primary antibody 25µL droplets of rabbit anti-PIP39 diluted 1:100 in incubation buffer (PBS, containing 0.1% Tween 20, 0.1% BSA, pH 7.3), for 2h at room temp. After washing, 4 x 10 min in incubation buffer, grids were incubated in a 1:1 mix of protein A (EMS - 25285), plus protein G (EMS - 25315) 10 nm gold, diluted 1:25 in incubation buffer for 2h at room temp. Grids were washed 2 x 10 min in PBS, containing 0.1% Tween 20, 0.1% BSA, pH 7.3, then 2 x 10 min in PBS, pH 7.3, then 2 x 10 min in Milli-Q water. Grids were stained in 2% uranyl acetate for 1min at room temp then washed 4 x 10 min in Milli-Q water and visualised on a Technai 12 at 120Kv.

**Live cell microscopy**

***Sample preparation for live cell microscopy***

For studying stumpy to procyclic form differentiation by live cell microscopy 25 µL of pH7.6, 0.6 M, filtered *cis*-aconitate was added to 2.5 ml of stumpy culture (4x10^6^/ml) to induce differentiation.

After 10 mins 1mL sample of the induced culture was taken to concentrate for the hydrogel fixation. Samples were spun for 10 min at 1400 rpm at 37C and 980 µL supernatants were removed after the spin cells were carefully resuspended in 20 µL culture media.

Immobilisation of living cells was performed with a two component hydrogel, consisting of 8-arm poly ethylene glycol (PEG) norborene and linear PEG-dithiol, essentially as described in (44). 4µl of hydrogel produced with TDB was mixed with a 2 µL solution containing 4x10^5^ differentiating stumpy cells and incubated 1 minute on ice. The mixture was transferred to a glass bottom cell culture dish (FluoroDish, FD35, World Precision Instruments) and allowed to polymerise at room temperature. Cells were viable in the hydrogel for at least 60 minutes (Glogger et al., 2017). In the experiments for this work, the hydrogel concentration was chosen so as to prevent translocation of cells, but still allow flagellar movement. As a control, 100 µL of the induced culture was taken and spun at the same speed and time as the immobilised sample. The cell pellet was carefully resuspended in 1.9 ml of warm TDB (2x105/ml) and was used to overlay the hydrogel droplet containing *cis*-aconitate induced stumpy cells.

Live imaging was performed with a Leica DMI6000B fully automated inverse microscope equipped with a 100x (NA1.4) oil immersion objective and a pco.edge SCMOS camera (PCO, Kelheim, Germany). Time series were recorded with 100 fps (100 ms resolution), which allowed the accurate detection of dynamic fluorescent signals in the cytoplasm of the living cells. Continuous videos were recorded switching between the fluorescence excitation and DIC settings. Due to the repetitive, periodic beating of the flagellum and cell movement of the immobilised trypanosome, the fluorescent and DIC images of corresponding phase shifted time series could be overlaid using Fiji (Fig. S1B, 0h)
