# Supplementary material for: Positional Dynamics and Glycosomal Recruitment of Developmental Regulators during Trypanosome Differentiation
Source: mBio. 2019 Jul 9;10(4):e00875-19. doi: 10.1128/mBio.00875-19 (PMC6747725; doi:10.1128/mBio.00875-19)
Supplement: TABLE S2 [file mBio.00875-19-st002.docx]

**Table S2: Antibodies used in the study**

| Antibody name | Animal raised in | Dilution used on Western Blot (W)  or in immuno fluorescence assays (IF) |
| --- | --- | --- |
| PIP39 | Rb | 1:750 W, 1:300 IF |
| BB2 | Ms | 1:5 W, 1:4 IF |
| EF1 | Ms | 1:7000 W |
| EP procyclin | Ms | 1:3000 W, 1:250 IF |
| gTIM | Ms | 1:500 IF |
| Aldolase | Ms | 1:250 IF |
| p67 | Ms | 1:1000 IF |
| REG 9.1 | Rb | 1:1000 IF |
| Alexa568 Red | Ms/Rb | 1:500 IF |
| Alexa488 Green | Ms/Rb | 1:500 IF |
| Licor Red Rb | Ms/Rb | 1:7500 W |
| Licor Green Ms | Ms/Rb | 1:7500 W |
